# Children’s Internet use and physical and psychosocial development

**DOI:** 10.3389/fpubh.2023.1163458

**Published:** 2023-06-08

**Authors:** Svetlana Novaković, Sanja Milenković, Marijana Srećković, Dušan Backović, Vladimir Ignjatović, Nataša Capo, Tamara Stojanović, Vladimir Vukomanović, Marija Sekulić, Jagoda Gavrilović, Katarina Vuleta, Vesna Ignjatović

**Affiliations:** ^1^Public Health Institute Požarevac, Požarevac, Serbia; ^2^Faculty of Medicine, Institute of Hygiene and Medical Ecology, University of Belgrade, Belgrade, Serbia; ^3^Department of Medical and Business-Technological, Academy of Professional Studies Šabac, Šabac, Serbia; ^4^Public Health Institute Šabac, Šabac, Serbia; ^5^Faculty of Medicine, University of Novi Sad, Novi Sad, Serbia; ^6^Department of Internal Medicine, Faculty of Medical Sciences, University of Kragujevac, Kragujevac, Serbia; ^7^Department of Cardiology, University Clinical Center Kragujevac, Kragujevac, Serbia; ^8^Department for Prevention of Rabies and Other Infectious Novi Sad, Pasteur Institute Novi Sad, Novi Sad, Serbia; ^9^Faculty of Philology and Arts, University of Kragujevac, Kragujevac, Serbia; ^10^Department of Nuclear Medicine, Faculty of Medical Sciences, University of Kragujevac, Kragujevac, Serbia; ^11^Department of Hygiene and Ecology, Faculty of Medical Sciences, University of Kragujevac, Kragujevac, Serbia; ^12^Department of Infectious Diseases, Faculty of Medical Sciences, University of Kragujevac, Kragujevac, Serbia

**Keywords:** Internet use, schoolchildren, obesity, sedentary behavior, vision, spine deformities, physical development, psychosocial development

## Abstract

**Introduction:**

Internet use (IU) commonly refers to sedentary lifestyle and may be addictive, especially among children. The aim of this study was to investigate the relationship between IU and some aspects of child physical and psychosocial development.

**Methodology:**

We conducted a cross-sectional survey by using a screen-time based sedentary behavior questionnaire and Strengths and Difficulties Questionnaire (SDQ)—among 836 primary school children in the Braničevo District. The children’s medical records were analysed for vision problems and spinal deformities. Their body weight (BW) and height (BH) were measured and body mass index (BMI) was calculated as BW in kilograms divided by BH in meters squared (kg/m^2^).

**Results:**

The average age of respondents was 13.4 (SD 1.2) years. The mean duration of daily Internet use and sedentary behavior was 236 (SD 156) and 422 (SD 184) minutes, respectively. There was no significant correlation between daily IU and vision problems (near sightedness, farsightedness, astigmatism, strabismus), and spinal deformities. However, daily Internet use is significantly associated with obesity (*p* < 0.001) and sedentary behavior (*p* = 0.01). There was significant correlation between emotional symptoms with total Internet usage time, and total sedentary score (*p* < 0.001 for both, *r* = 0.141 and *r* = 0.132, respectively). There was a positive correlation between the total sedentary score of children and hyperactivity/inattention (*r* = 0.167, *p* < 0.001), emotional symptoms (*r* = 0.132, *p* < 0.001), and conduct problems (*r* = 0.084, *p* < 0.01).

**Conclusion:**

In our study, children’s Internet use was associated with obesity, psychological disturbances and social maladjustment.

## 1. Introduction

The Internet has been increasingly used for obtaining information, entertainment, socialization and education. The Internet overuse among children may lead to an increase in sedentary habits and health problems such as the following: obesity, poor posture, psychological disturbances and eye disorders (1–3). The American Academy of Pediatrics (AAP) recommends that the time spent in front of screens on any digital device should not be longer than 2 h per day (4). The data obtained from the study, conducted in seven European countries including: Germany, Greece, Iceland, the Netherlands, Poland, Romania, and Spain, indicate an association between Internet use and overweight/obesity among adolescents (5). Asian countries also encounter similar problems with children’s obesity, which may be connected to Internet addiction (6–8).

Internet overuse has been associated with developing cumulative musculoskeletal disorders (MSDs) and computer vision syndrome (CVS). MSDs are often characterized with muscle fatigue and discomfort, leading to pain (9, 10). A previous study on children confirmed the fact that there was an association between computer use and the occurrence of MSDs (11). Indeed, heavy use of digital devices is also associated with the new ophthalmic syndromes: “digital eye strain” (12) and “video game vision” (13). The findings of a recent study conducted among schoolchildren in Qatar revealed a higher prevalence of vision disorders among frequent Internet users and television viewers (14). However, The Collaborative Longitudinal Evaluation of Ethnicity and Refractive Error (*CLEERE*) Study Group showed that near work activities in myopic-children did not differ from the emmetropic ones (15). Sherwin et al. performed a meta-analysis and found that outdoor time might be protective against myopia (16). Epidemiological studies have indicated that musculoskeletal and visual problems in childhood might be tracked on into adulthood (17). Excessive Internet use (IU) has been associated whit adverse psychosocial development and functioning, including social isolation and impaired social skills among adolescents (18–20). Internet use has also been associated with attention deficit, hyperactivity disorder (21) and major depressive episodes (22).

The aim of our study was to investigate the relationship between excessive IU and some aspects of child physical and psychosocial development.

## 2. Materials and methods

### 2.1. Sampling

This cross-sectional study was performed on a sample of 836 children (response rate 98.6%) from two urban and two rural primary schools in the Braničevo district, Serbia. The study was carried out from April 2019 to May 2019.

### 2.2. Questionnaire

The examination of the connection between Internet use and physical and psychosocial characteristics of schoolchildren was designed as a cross-sectional observational study. All students from the fifth to the eighth grade in two villages and two city primary schools were included. All students with normal psychological development are included.

Before the start of the research, ethical approval was obtained from the management of the Institute of Public Health Požarevac. The survey was voluntary, and the respondents received detailed instructions from the research team how to fill out the questionnaire. Students and parents were informed about the methodology and purpose of the research, as well as the fact that their data will be kept confidential and used exclusively for research purposes. The questionnaire was anonymous, and additional confidentiality data is guaranteed by placing them in unmarked envelopes and boxes.

The children completed a questionnaire at home with parents. The questionnaire included three parts:

Socio-demographic data, Screen time-based sedentary behavior questionnaire, and Strengths and Difficulties Questionnaire. The third part of the Questionnaire consisted of the standardized Strengths and Difficulties Questionnaire (SDQ-Srp), which was completed by parents. The designed sedentary behaviors questionnaire was based on a previous study conducted in Belgium and Spain (23).

Children reported their habitual time devoted to several sedentary behaviors: (1) TV watching, (2) computer games, (3) console (video) games, (4) Internet for non-study reasons (hobbies), (5) Internet for study reasons, and (6) study time (out of scholar schedule). In a closed type of questionnaire, there were the six categories, following daily time: (I) 0 min, (II) >0–30 min, (III) >30–60 min, (IV) >60–120 min, (V) >120–180 min, (VI) >180–240 min and (VI) >240 min. Weekly time was calculated taking the median value in a selected category, and applying this formula: [(weekdays × 5) + (weekend × 2)]/7. A total sedentary score was obtained by summing up the time reported in each category.

The SDQ was used to assess participants’ emotional and psychosocial adjustment (24). The SDQ includes 25 questions with answers scored from 0 to 2. The scales include: (1) Emotional symptoms subscale; (2) Conduct problems subscale; (3) Hyperactivity/inattention subscale; (4) Peer relationships problem subscale; (5) Prosocial behavior subscale. We excluded the Prosocial Scale, and the sum (range 0–40) of the remaining components constituted the Total Strengths and Difficulties score.

### 2.3. Anthropometry and medical records

Children’s medical records were analyzed for vision problems and spine deformities. Their body weight (BW) and height (BH) were measured and body mass index was calculated as BW in kilograms divided by squared BH in meters. Anthropometric measurements were carried out at Public Health Institute Požarevac. Doctors and medical technicians with appropriate education performed the measurements. Body height was measured with an anthropometer and expressed in centimeters, with an accuracy of 1 cm. Body height was measured with subjects standing on a flat surface with their heels together. Body mass was measured with a medical decimal scale (Detecto 2,371 s) and was expressed in kilograms. During the measurement, the subjects were in underwear, without shoes.

Body Mass Index (BMI) is a number calculated from weight and height using WHO AnthroPlus software (25).

### 2.4. Statistical analysis

Statistical analysis was performed using SPSS Statistics for Windows, Version 20.0. Descriptive statistics included proportions and percentages as summary statistics of the differences between the groups. In the end, the groups are compared based using proportion and percentages. The mean and SD were used to summarize distribution of numerical variables. Inferential statistics analysis was performed using Student *t*-test. Pearson correlation analysis was used to determine the correlation between continuous variables. In all statistical tests, *p*-value < 0.05 was considered as significance.

## 3. Results

Our study included 836 children, 520 from urban and 316 from rural schools. There were 431 boys and 405 girls who participated in the study. The age range was 11–16, with the average age of 13.4 ± 1.2 years. There were 175 fifth grade students, 250 sixth grade students, 204 seventh grade students and 200 eighth grade students.

The average age when children started with Internet use was 8.3 ± 2.24 years. Smartphones, desktop computers and laptops were used by 92.2, 76.3, and 40.6% of children, respectively. The purpose of IU was mostly electronic communication (65%), than electronic games (48%), and education (45%). The average daily duration of Internet use and sedentary behavior was 236 ± 156 and 422 ± 184 min.

This study indicated no significant differences according to the average daily use of electronic devices between children from rural and urban communities (Table 1). Daily duration of IU and total sedentary score were higher among boys compared to girls. The boys used Internet mainly for socialization and entertainment, while the Internet use among girls was mainly for educational purposes (Table 1).

**Table 1:** Purpose and average daily use of electronic devices among the studied children according to dwelling place and gender.

Variables (minutes per day)	Mean ± SD	Mean ± SD	p*
	Dwelling place		
	Rural areas	Urban areas	
Playing computer games	60.4 ± 72.9	58.6 ± 73.7	0.624
Playing video game console	40.8 ± 61.6	43.3 ± 63.9	0.541
Playing mobile games	101.0 ± 117.6	101.8 ± 123.7	0.897
IU as a hobby	85.7 ± 70.2	89.7 ± 70.6	0.389
IU for educational purposes	53.3 ± 47.5	43.2 ± 44.3	<0.001
Other purpose the Internet usage	138.4 ± 95.1	132.8 ± 90.4	0.500
Total Internet usage time	239.6 ± 155.4	234.4 ± 155.8	0.484
Total sedentary score	423.3 ± 192.5	421.8 ± 179.4	0.857
	Gender		
	Girls	Boys	
Playing computer games	21.9 ± 45.4	99.0 ± 76.5	<0.001
Playing video game console	17.8 ± 40.7	68.4 ± 71.4	<0.001
Playing mobile games	39.5 ± 74.8	167.5 ± 126.7	<0.001
IU as a hobby	103.5 ± 72.4	71.9 ± 64.5	<0.001
IU for educational purposes	52.3 ± 49.1	41.3 ± 41.2	<0.001
Other purpose the Internet usage	155.5 ± 94.0	112.9 ± 84.9	<0.001
Total Internet usage time	194.8 ± 129.7	280.7 ± 168.3	<0.001
Total sedentary score	386.2 ± 168.5	461.0 ± 192.5	<0.001

*Student *t*-test.

The prevalence of wearing eyeglasses in the studied children was 16.9%, with an average age at diagnosis refractive errors of 8.7 ± 3.1 years. The most frequent refractive anomalies were nearsightedness and farsightedness (9.6 and 5%, respectively). There was no significant relationship between daily Internet use and refractive errors (Table 2). The study findings indicated that the prevalence of musculoskeletal deformities of spine and poor postures among children was 4.8 and 12.8%, respectively. There was no significant relationship between daily Internet use and poor body posture and spine deformities among children (Table 2).

**Table 2:** Associations between Internet use and sedentary score and wearing glasses, refractive errors, poor body posture, spine deformities and the prevalence of obesity among the studied children.

Variables (minutes per day)	Mean ± SD	Mean ± SD	p*
	Wearing glasses		
	Yes	No	
Total Internet usage time	223.3 ± 145.1	239.0 ± 157.6	0.35
Total sedentary score	397.3 ± 172.7	427.5 ± 186.2	0.06
	Refractive errors		
	Yes	No	
Total Internet usage time	237.7 ± 157.2	225.3 ± 138.4	0.61
Total sedentary score	426.3 ± 184.2	397.9 ± 167.6	0.11
	Poor body posture		
	Yes	No	
Total Internet usage time	257.7 ± 166.0	232.2 ± 152.1	0.20
Total sedentary score	432.3 ± 181.3	419.9 ± 181.9	0.62
	Spine deformities		
	Yes	No	
Total Internet usage time	204.3 ± 146.5	237.0 ± 154.5	0.14
Total sedentary score	381.0 ± 164.1	423.3 ± 182.4	0.13
	Obesity		
	Yes	No	
Playing computer games	104.1 ± 88.1	56.7 ± 71.8	<0.001
Playing video game console	62.9 ± 78.6	40.9 ± 62.2	0.105
Playing mobile games	168.8 ± 147.6	97.5 ± 119.7	<0.001
Internet use as a hobby	91.7 ± 74.1	89.7 ± 70.2	0.992
Internet use for educational purposes	48.5 ± 40.3	47.4 ± 45.8	0.432
Other purpose the Internet usage	140.2 ± 82.8	136.7 ± 92.4	0.607
Total Internet usage time	309.3 ± 170.4	233.7 ± 152.3	<0.001
Total sedentary score	488.1 ± 194.6	421.4 ± 181.5	0.012

*Student *t*-test.

The prevalence of obesity among the studied children was 6.9%. We found a significant association between the duration of total Internet usage time and total sedentary score with obesity among primary school students (*p* < 0.001 and *p* < 0.05, respectively; Table 2).

According to the children’s answers from the questionnaires, 720 respondents (89.4%) have never visited the psychologist and/or psychiatrist, 23 (2.8%) considered the visit, while 88 (10.6%) went to see the psychologist and/or psychiatrist one or more times.

Concerning the total score of difficulties 4.6% of children had a borderline value, and 2.4% an abnormal total value. Hyperactivity-inattention, emotional symptoms, and conduct problems were correlated with daily IU. There was significant correlation between emotional symptoms with total Internet usage time, and total sedentary score (*p* < 0.001 for both, *r* = 0.141 and *r* = 0.132 respectively); when Internet usage time is higher and thus total sedentary score, emotional symptoms were more pronounced (Table 3).

**Table 3:** Correlations between daily duration of Internet use and psychosocial distress among the studied children.

Variables (minutes per day)	Emotional symptoms	Conduct problems	Hyperactivity/inattention	Peer relationships problems	Prosocial behavior	Total Strengths and Difficulties score
Playing computer games	−0.030	0.089*	0.216**	0.071*	−0.072*	0.111**
*p*	0.405	0.012	<0.001	0.045	0.040	0.002
Playing video game console	0.027	0.072*	0.177**	0.047	−0.090*	0.102**
*p*	0.440	0.043	<0.001	0.184	0.011	0.004
Playing mobile games	−0.002	0.116**	0.245**	0.061	−0.097**	0.137**
*p*	0.965	<0.001	<0.001	0.085	0.006	<0.001
IU as a hobby	0.176**	0.060	0.062	0.039	−0.093**	0.113**
*p*	<0.001	0.088	0.081	0.273	0.009	0.002
IU for educational purposes	0.082*	−0.094**	−0.051	0.064	0.046	−0.005
*p*	0.021	0.008	0.149	0.069	0.194	0.899
Other purpose the Internet usage	0.195**	0.016	0.030	0.061	−0.054	0.098**
*p*	<0.001	0.653	0.394	0.085	0.127	0.006
Total Internet usage time	0.141**	0.119**	0.201**	0.090*	−0.134**	0.184**
*p*	<0.001	<0.001	<0.001	0.011	<0.001	<0.001
Total sedentary score	0.132**	0.084**	0.167**	0.066	−0.122**	0.147**
*p*	<0.001	0.019	<0.001	0.064	<0.001	<0.001

**Correlation is significant at the 0.01 level, and * at the 0.05 level.

There was a positive correlation between the total sedentary score of children and hyperactivity/inattention (*r* = 0.167, *p* < 0.001), emotional symptoms (*r* = 0.132, *p* < 0.001), and conduct problems (*r* = 0.084, *p* < 0.01). We have also found significant correlation between hyperactivity/inattention and Internet usage time and playing games (Table 3). Total strengths and difficulties score had positive significant correlation with playing mobile games (*p* < 0.001, *r* = 0.137), total Internet usage time (*p* < 0.001, *r* = 0.184) and total sedentary score (*p* < 0.001, *r* = 0.147). Furthermore, schoolchildren’s prosocial behavior was negatively correlated with the use of the Internet (*p* < 0.001, *r* = −0.134), which means that Internet use was associated with negative prosocial behavior (Table 3).

## 4. Discussion

We have found no significant relationship between daily Internet use and vision problems (shortsightedness, farsightedness, astigmatism, and strabismus) and spine deformities. However, there is a significant association between daily duration of Internet use and children’s obesity. We also detected and reported a positive correlation between the duration of children’s Internet use and hyperactivity-inattention, emotional symptoms, conduct problems and social maladjustment.

Our analysis has shown that boys use Internet more frequently than girls do and that is congruent with previous studies (26). Daily sedentary time was also longer among boys compared to girls. However, in a study conducted in Catalonia, boys scored higher than girls for physical activity (27). Average daily sedentary time was longer in our research compared with the previous studies (28, 29). These findings highlight the need for domain-focused strategies to decrease sedentary behavior. Current activity guidelines (4) recommend no more than 2 h per day of recreational screen time (i.e., watching TV, DVDs, or videos, playing video games, and computer use).

In our study, there was an association found between children’s Internet use and sedentary time with obesity. The research conducted in Hunan (China) also found that middle school students with Internet addiction had a significantly higher prevalence of obesity compared to those without Internet addiction (30). Establishing a comprehensive management program that emphasizes behavioral modification is important for prevention of childhood obesity (31). Therefore, interventions should target reducing after-school sedentary behaviors and increasing physical activity. Studies in developed countries reported that only 33% of adolescents fulfil a recommended goal of at least 60 min of physical activity per day (32). Nearly half of the children in our study did not exercise any sport, which is similar to the situation among schoolchildren in other parts of Serbia (33) and in India (34). Sedentary behavior can negatively affect mental and cognitive health in children and adolescents with obesity (35).

Hence, in our study, there was no significant association between the Internet use and visual impairments, and this finding is in contrary to the report of the American Optometric Association that an individual who exceeds 2 h of computer use daily is at risk for developing computer vision syndrome (36). However, the studies conducted among children in Singapore found no evidence of refractive anomalies associated with increased computer viewing (37, 38). Increased time outdoors may be an effective measure in preventing the onset of myopia (39).

The prevalence of children’s spinal deformities and bad posture, as reported in our study, was 4.8 and 12.8%, respectively. Data from the previous review indicated the prevalence of 0.47–5.2% for children’s idiopathic scoliosis (40). In our research, there was no significant correlation found between daily Internet use and bad posture or spinal deformities. This finding is contrary to the reports confirming that musculoskeletal discomfort among American children may be associated with computer use (11). Additionally, a recent longitudinal study showed that more than 2 h per day of TV watching and computer use were associated with decreased musculoskeletal fitness (41).

The present study indicates that a significant number of adolescents suffer from emotional and psychosocial maladjustment, as assessed by the total SDQ score. In addition, Kormas et al. (19) and Critselis et al. (26) reported the correlation between Internet use and compromised psychosocial well-being. Few of previous studies have also showed that the increased Internet use is associated with an enhanced likelihood of hyperactivity and conduct problems among adolescents (3, 42). However, on the other hand, other results of other studies on prosocial and antisocial behavior associated with the Internet use are still controversial (43, 44).

There is compelling evidence showing that sitting by personal computer too much may impair physical, mental and cognitive health in children and adolescents (34, 45). In relation to our own contribution to the field, we found a statistically significant association between daily Internet use (sedentary lifestyle) and obesity, hyperactivity-inattention, emotional symptoms, conduct problems and social maladjustment.

We believe that greater involvement of multidisciplinary state institutions is necessary in overcoming the problem of excessive use of smart devices. Teachers, parents and children should be educated about the importance of setting clear limits on the time spent on smartphone. Parents and teachers, also, should be educated in order to detect possible problems in children’s behavior early. In addition, the state should enable fostering healthy lifestyles, by increasing the availability and number of cultural and sports content. Parents and teachers should with a lot of love, understanding and respect, help children to find a balance between the time spent on smart devices and other activities. Children should reduce the use of the Internet and give priority to creative play without the presence of smart devices, to play sports and to spend more time in nature.

## 5. Conclusion

In our study, the children’s Internet use and sedentary behavior were associated with obesity, but not with vision problems or spinal deformities. In addition, we found a positive significant relationship between children’s Internet use and hyperactivity, emotional problems and social difficulties. It can be expected that the reduction in children’s daily Internet use might have a positive effect on maintaining their mental health and normal body weight as well.

Since this research was done before the COVID-19 pandemic, it would be very important to repeat the study after the end of the pandemic and compare the results obtained.

## 6. Limitations of study

A precise electronic system was not used to measure time in front of the screen. The measurement is based on the assessment of parents and children about the amount of time spent using the smart devices. The parents together with the children did time estimation. Subjective assessment of children’s time in front of the screen could not be excluded when filling out the SDQ questionnaire.

Possibility of confounding phenomena, because both excessive use of social networks and obesity can be consequences of the same thing, which is a sedentary lifestyle.

## Data availability statement

The original contributions presented in the study are included in the article/supplementary material, further inquiries can be directed to the corresponding author.

## Ethics statement

Ethical approval was provided by the Institute for Public Health Pozarevac. Written informed consent to participate was provided by the participants legal guardian.

## Author contributions

All authors listed have made a substantial, direct, and intellectual contribution to the work and approved it for publication.

## Conflict of interest

The authors declare that the research was conducted in the absence of any commercial or financial relationships that could be construed as a potential conflict of interest.

## Publisher’s note

All claims expressed in this article are solely those of the authors and do not necessarily represent those of their affiliated organizations, or those of the publisher, the editors and the reviewers. Any product that may be evaluated in this article, or claim that may be made by its manufacturer, is not guaranteed or endorsed by the publisher.
